# Antimicrobial resistance profiles in bacterial species isolated from fecal samples of free-ranging long-tailed macaques (*Macaca fascicularis*) living in Lopburi Old Town, Thailand

**DOI:** 10.14202/vetworld.2020.1397-1403

**Published:** 2020-07-22

**Authors:** Duangjai Boonkusol, Suporn Thongyuan, Nantana Jangsuwan, Pornchai Sanyathitiseree

**Affiliations:** 1 Department of Biology, Faculty of Science and Technology, Thepsatri Rajabhat University, Lopburi, Thailand; 2 Department of Veterinary Public Health, Faculty of Veterinary Medicine, Kasetsart University, Nakhon Pathom, Thailand; 3 Department of Large Animal and Wildlife Clinical Science, Faculty of Veterinary Medicine, Kasetsart University, Nakhon Pathom, Thailand

**Keywords:** antibiotic, drug, monkey, resistant, susceptible

## Abstract

**Background and Aim:**

At present, increasing in long-tailed macaques (*Macaca fascicularis*) population in Lopburi old town caused several problems in its community, in particular with sanitation problem. The present study aimed to explore species distribution and antimicrobial resistance patterns in bacteria isolated from feces of the free-ranging long-tailed macaques (*Macaca fascicularis*) in Lopburi Old Town, Thailand.

**Materials and Methods:**

Fresh fecal samples were collected from October 2018 to July 2019 from seven troops of macaques. Bacterial colonies were identified based on Gram stain and standard biochemical techniques. Sensitivity toward eight different antibiotics, including amoxicillin, amoxicillin-clavulanate, cephalexin, clindamycin, doxycycline, enrofloxacin, erythromycin, and gentamicin, was analyzed using the disk diffusion method.

**Results:**

A total of 1050 fecal samples were collected. Five unique bacterial species were identified, including *Escherichia coli*, *Enterobacter* spp., *Proteus* spp., *Salmonella* Group B, and *Citrobacter* spp. in 100%, 25.71%, 18%, 1.71%, and 0.57% of the fecal specimens, respectively. Among 70 distinct isolates of *E*. *coli*, 63 (93%) were resistant to multiple drugs, including amoxicillin, cephalexin, clindamycin, and erythromycin; one isolate (6%) was resistant to clindamycin only. Furthermore, 17 isolates (94%) of *Salmonella* Group B were resistant to both clindamycin and erythromycin. Five of the six *Citrobacter* spp. isolates (83%) were also multidrug-resistant (to cephalexin, clindamycin, and erythromycin); the one remaining *Citrobacter* spp. isolate (6%) was resistant to both clindamycin and erythromycin. However, a high percentage of *E. coli*, *Salmonella* Group B and *Citrobacter* spp. remained susceptible to amoxicillin-clavulanate, enrofloxacin, and doxycycline.

**Conclusion:**

Our findings provide the basic information for the selection of empirical therapy and for the evaluation of the scale of antibiotic resistance associated with macaques living in Lopburi Old Town.

## Introduction

Long-tailed macaques (*Macaca fascicularis*) living in the Old Town of Lopburi Province, Thailand, which is the site of the Phra Prang Sam Yod Khmer shrine, are recognized as important symbols of Lopburi and have attracted visitors from around the world. The macaques live freely in the Old Town area; the buildings and ancient structures are their habitats. The macaques are most likely to be found in the downtown areas, including the roadsides and the railway station, as they are frequently fed by local inhabitants and by tourists [1]. Given the current urban ecosystem, the macaques are almost entirely dependent on food provided by humans for their ongoing survival. Tourists are encouraged to provide food for the macaques by placing it on the ground or by hand feeding. They are typically provided with human-style foodstuffs, including steamed rice, vegetables, fruits, eggs, candies, jellies, and yogurt-based and other beverages. Furthermore, macaques have learned to acquire food by snatching items directly from humans and by scavenging in the trash; some macaques go to great lengths to obtain human food and terrorize people in their homes. These behaviors serve to maximize human-animal interactions in this area of the world. The previous studies have revealed the diversity of gastrointestinal bacteria in members of the animal kingdom; among these, *Salmonella* spp. and *Escherichia coli* were identified in fecal samples from wild birds [2,3], wild animals [4,5], wild Taihangshan macaques [6], and captive wildlife [7]; these studies have shown that diet strongly influences the composition of the gastrointestinal microbiome [8]. Humans that interact with wild and domestic animals can have similar gastrointestinal bacteria, presumably associated with their origins from common environmental sources [9-11]. Furthermore, antimicrobial agents play a critical role in the treatment of bacterial infections in both human and veterinary medicine [12]. Fecal bacteria identified in wild animal species are frequently resistant to the older, naturally occurring antibiotics because of their frequent contact with humans and domestic animals, as well as with human landscapes and environments [8,13-17]. The abundance of resistant microorganisms detected tends to be associated with proximity to human settlements [18-22]. Likewise, inadequately treated wastes from humans and livestock; the latter often dosed with antimicrobial drugs, have been assumed to be among the main sources of antimicrobial resistance in the wild [23]. There are currently a wide variety of multiresistant genes associated with multiresistant non-pathogenic *E. coli* strains detected in humans, animals, and food products; normal flora may serve as both acceptors and donors of transmissible antimicrobial resistance [24].

Antibiotic resistance in wildlife represents a potential public health threat [25]; as such, it will be critical to have some understanding of the bacterial pathogens and antibiotic resistance mechanisms involved so that disease outbreaks might be contained and treated. There is certainly a high likelihood of human-macaque disease transmission in the heavily traveled regions of Thailand. However, at this time, there is only a limited amount of information on endogenous bacterial species and antibiotic resistance profiles that can be isolated from fecal specimens from long-tailed macaques in Thailand.

Therefore, this study aimed to explore the species distribution and antimicrobial resistance profiles of bacteria isolated from the free-ranging long-tailed macaques (*M. fascicularis*) living in Lopburi Old Town, Thailand.

## Materials and Methods

### Ethical approval

This study does not need ethical approval. However, all applicable international and national guidelines for the care and use of animals were followed.

### Study area

**Figure-1:**
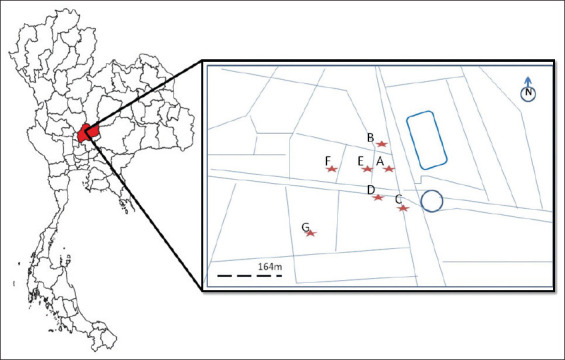
Map showing the locations of fecal samples collected from long-tailed macaques in Lopburi Old Town, Thailand. A=Prang Sam Yot Khmer shrine, B=Malai Rama Theater, C=Van station, D=Chayowanich building, E=Muang Thong hotel, F=Manora Market, G=Seng Heng building.

Fecal samples were collected monthly for 10-month period during October 2018-July 2019. Seven troops of long-tailed macaques (*M. fascicularis*) living in downtown of Lopburi Province which covered area around 0.09 km^2^ were observed. According to the previous studied, the estimated total population of macaques in Old Town Lopburi was 2080 individuals which distributed into seven locations including 700 individuals at Prang Sam Yot Khmer shrine (A: 14°48’10”N 100°36’50”E), 400 individuals at Malai Rama Theater (B: 14°48’5” N, 100° 37’1” E), 100 individuals at Van station (C: 14°48’8” N, 100°36’55” E), 300 individuals at Chayowanich building (D: 14°48’10” N, 100°36’54” E), 150 individuals at Muang Thong hotel (E: 14°48’10”N, 100°36’53” E), 80 individuals at Manora Market (F: 14°48’9” N, 100°36’46” E), and 350 individuals at Seng Heng building (G: 14°48’3”N, 100°36’49” E) [1]. The mean annual temperature is 28.2°C (24.0-33.8°C), and the annual precipitation amounts to 1125 mm. The locations of seven troops are shown in Figure-1.

### Fecal sample collection

A total of 105 fecal samples were collected each month from October 2018 to July 2019; this study design was based on a calculation of estimated absolute precision ±4% with a 95% confidence interval and an expected recovery of bacteria from 18% of the specimens collected, as per findings in our pilot study. Sample size from each troop was determined with probability proportional to the number of macaques in the given population. We observed the macaques at seven different locations between the hours of 6:00 and 8:00. Fecal samples were collected immediately after defecation. To avoid environmental contamination, fecal samples were picked from the center part of the stool lump and stored in sterile polyethylene bags that were clearly labeled with the location; samples were held on ice packs for no more than 3 h until refrigeration was available.

### Bacterial isolation and identification

Three grams of fecal sample were mixed with normal saline and subjected to centrifugation; the material remaining was used to inoculate 5 ml of buffered peptone water (Oxoid Ltd., Basingstoke, Hampshire, England), which was then incubated at 37°C for 24 h. Samples were streaked onto individual selective media for bacterial isolation and identification, including blood agar (Oxoid Ltd., Basingstoke, Hampshire, England), MacConkey agar (HiMedia Laboratories Pvt. Ltd., Mumbai, India), and Salmonella Shigella agar (HiMedia Laboratories Pvt. Ltd., Mumbai, India); streaked agar plates were incubated at 37°C for 24 h. Single colonies were subcultured on blood agar and MacConkey agar. Colonies obtained in MacConkey agar were identified as *Enterobacteriaceae* or *Pseudomonadaceae* using the oxidase test (Becton Dickinson and Company, MD, USA). Colonies that were oxidase negative (*Enterobacteriaceae*) were evaluated on triple sugar iron (TSI) agar (HiMedia Laboratories Pvt. Ltd., Mumbai, India) before performing an IMViC test (Oxoid Ltd., Basingstoke, Hampshire, England), sulfide indole motility (Oxoid Ltd., Basingstoke, Hampshire, England), lysine (HiMedia Laboratories Pvt. Ltd., Mumbai, India), urea (HiMedia Laboratories Pvt. Ltd., Mumbai, India), and arabinose (HiMedia Laboratories Pvt. Ltd., Mumbai, India) to confirm the species of the Gram-negative bacterial isolate. Colonies positive for the use of lysine/arabinose, H_2_S, and urea in the TSI test were verified as *Salmonella* spp . by an agglutination test. The identification of bacteria by light microscopy based on morphology, colony size, and staining characteristics was conducted according to Christopher and Bruno [26].

### Antimicrobial susceptibility

Isolates representing four bacterial species, including *E. coli*, *Enterobacter* spp., *Salmonella* spp., and *Citrobacter* spp., were tested for susceptibility to eight antibiotics, including amoxicillin, amoxicillin-clavulanate, cephalexin, clindamycin, doxycycline, enrofloxacin, erythromycin, and gentamicin using the disk diffusion method (BBL disks on Mueller-Hinton agar, Becton Dickinson and Company, MD, USA). The diameters of the zones of inhibition were measured in millimeter units with a caliper; results were interpreted according to guidelines from the Clinical and Laboratory Standards Institute [27].

### Statistical analysis

Data were analyzed using NCSS for Windows version 10.0 (NCSS, LLC. Kaysville, Utah, USA). Distribution of bacterial species isolated and antimicrobial sensitivity profiles were ­evaluated using descriptive statistics and presented as frequencies and percentages.

## Results

We collected a total of 1050 fecal samples from seven sites; 350 samples were collected from Location A, 200 from Location B, 50 from Location C, 150 from Location D, 80 from Location E, 40 from Location F, and 180 samples from Location G. The seven sites at which fecal specimens were collected are shown in Figure-1.

### Distribution of bacterial species

**Figure-2:**
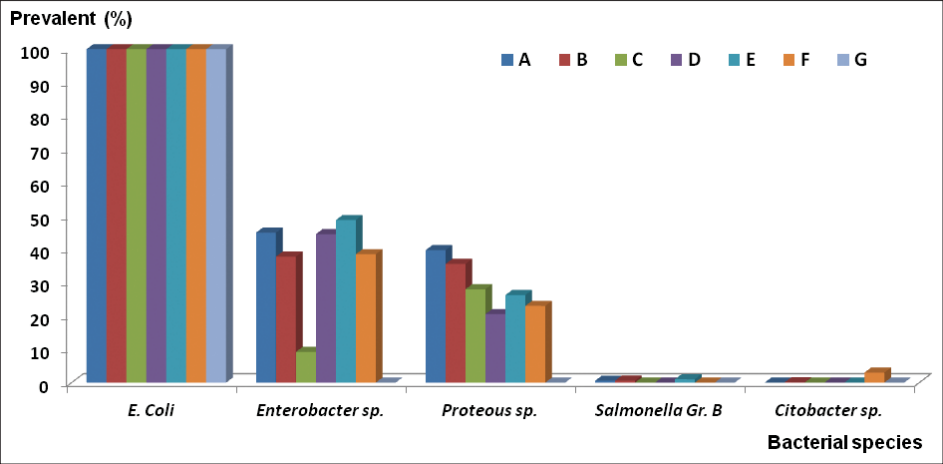
Distribution of bacterial species isolated from seven locations of Lopburi Old Town. A=Prang Sam Yot Khmer shrine, B=Malai Rama Theater, C=Van station, D=Chayowanich building, E=Muang Thong hotel, F=Manora Market, G=Seng Heng building.

The distribution of bacterial species isolated from fecal samples of free-ranging long-tailed macaques living in Lopburi Old Town is provided in Table-1. Four bacterial species were identified in the 1050 specimens, including *E. coli*, *Enterobacter* spp., *Proteus* spp., *Salmonella* Group B, and *Citrobacter* spp., which were isolated from 100%, 25.71%, 18%, 1.71%, and 0.57% of the specimens, respectively. Gram-negative bacteria, family Enterobacteriaceae, including *E. coli* and *Enterobacter* spp., were identified in all specimens from every location (Figure-2); by contrast, *Citrobacter* spp. were identified in four samples that were collected from the Don Manora market (Location F) in November 2018 and at two samples collected from Locations B and F in December 2018. *Salmonella* Group B was identified in 20 samples collected from October 2018 to December 2018.

**Table-1:** Distribution of bacterial species found in 1050 fresh fecal samples collected from free-ranging long-tailed macaques living in Lopburi Old Town between October 2018 and July 2019.

Month year	No. of sample	*E. coli*		*Enterobacter* spp.		*Proteus* spp.		*Salmonella* Group B		*Citrobacter* spp.	
				
		No.	%	No.	%	No.	%	No.	%	No.	%
October 2018	105	105	100	40	38.09	25	23.81	5	4.76	0	0
November 2018	105	105	100	15	14.29	0	0	8	9.52	3	2.86
December 2108	105	105	100	14	13.33	0	0	5	4.76	3	2.86
January 2019	105	105	100	13	12.38	0	0	0	0	0	0
February 2019	105	105	100	12	11.43	0	0	0	0	0	0
March 2019	105	105	100	17	16.19	26	24.76	0	0	0	0
April 2019	105	105	100	29	27.62	27	25.71	0	0	0	0
May 2019	105	105	100	40	38.10	35	33.33	0	0	0	0
June 2019	105	105	100	40	38.10	35	33.33	0	0	0	0
July 2019	105	105	100	50	47.62	40	38.10	0	0	0	0
Total	1050	1050	100	270	25.71	188	18.00	18	1.71	6	0.57

### Antimicrobial susceptibility profiles

Antibiotic susceptibility testing was conducted for *E. coli*, *Salmonella* Group B, and *Citrobacter* spp. (Table-2); full antibiotic resistance profiles are shown in Table-3.

**Table-2:** Antibiotic resistance patterns among *E. coli, Salmonella* Group B, and *Citrobacter* spp. from fecal samples of free-ranging long-tailed macaques living in Lopburi Old Town.

Bacterial species	*E. coli* (n=70)			*Salmonella* Group B (n=18)			*Citrobacter* spp. (n=6)		
			
Susceptibility/antibiotics	R n (%)	I n (%)	S n (%)	R n (%)	I n (%)	S n (%)	R n (%)	I n (%)	S n (%)
Amoxicillin	66 (94)	4 (6)	0	0	1 (6)	17 (94)	0	3 (50)	3 (50)
Amoxy+Clav	0	7 (10)	63 (90)	0	1 (6)	17 (94)	0	0	6 (100)
Cephalexin	70 (100)	0	0	0	2 (11)	16 (89)	5 (83)	1 (17)	0
Clindamycin	68 (97)	2 (3)	0	18 (100)	0	0	6 (100)	0	0
Doxycycline	0	4 (6)	66 (94)	0	2 (11)	16 (89)	0	1 (17)	5 (83)
Enrofloxacin	0	2 (3)	68 (97)	0	0	18 (100)	0	0	6 (100)
Erythromycin	70 (100)	0	0	17 (94)	1 (6)	0	6 (100)	0	0
Gentamicin	0	7 (10)	63 (90)	0	1 (6)	17 (94)	0	1 (17)	5 (83)

R=Resistant, I=Intermediate, S=Susceptible; Amoxy+Clav=Amoxicillin/clavulanate, *E. coli=Escherichia coli*

**Table-3:** Antibiotic resistance profiles among *E. coli, Salmonella* Group B, and *Citrobacter* spp. isolated from fecal samples of long-tailed macaques at Lopburi Old Town.

Resistant profiles	*E. coli* (n=70)	*Salmonella* Group B (n=18)	*Citrobacter* spp. (n=6)
Single resistant			
Clindamycin		1 (6)	
Double resistant			
Cephalexin-erythromycin	1 (1.4)		
Clindamycin-erythromycin		17 (94)	1 (7)
Multidrug resistant			
Amoxy-Ceph-Erythro	1 (1.4)		
Ceph-Clinda-Erythro	3 (4.2)		5 (83)
Amoxy-Ceph-Clinda-Erythro	65 (93)		

Amoxy=Amoxicillin, Ceph=Cephalexin, Clinda=Clindamycin, Erythro=Erythromycin, *E. coli=Escherichia coli*

Among all *E. coli* obtained from fecal samples, 70 of 1050 isolates were selected semi-randomly (one specimen isolated from each location during the 10-month period) for antibiotic susceptibility testing. Most of the isolates were susceptible to amoxicillin-clavulanate, doxycycline, enrofloxacin, and gentamicin; however, antibiotic resistance was also observed. All isolates were resistant to cephalexin and erythromycin, 68 (97%) were resistant to clindamycin, and 66 (94%) were resistant to amoxicillin. Double and multidrug-resistant profiles were observed in 1 (1.4%) and in 69 (98.6%) of isolates, respectively. Most of the isolates (93%) were resistant to multiple drugs, including amoxicillin, cephalexin, clindamycin, and erythromycin.

Among the 18 *Salmonella* Group B isolates, all were susceptible to enrofloxacin; 94% were susceptible to amoxicillin, amoxicillin-clavulanate, and gentamicin; and 89% were susceptible to cephalexin and doxycycline. Furthermore, 18 (100%) were resistant to clindamycin, and 17 (94%) were resistant to erythromycin; 17 (94%) of the isolates were resistant to two drugs (clindamycin and erythromycin), whereas only 1 isolate (6%) was resistant to clindamycin alone.

Antibiotic susceptibility testing was also conducted on six isolates identified as *Citrobacter* spp. All (100%) of *Citrobacter* isolates were susceptible to amoxicillin-clavulanate and enrofloxacin, 83% were susceptible to doxycycline and gentamicin, whereas only 50% were susceptible to amoxicillin. All isolates were resistant to clindamycin and erythromycin, and 5 isolates (83%) were resistant to cephalexin. Five isolates (83%) were resistant to multiple drugs (cephalexin-clindamycin-erythromycin), whereas only 1 isolate (7%) was identified as double drug-resistant (clindamycin-erythromycin).

## Discussion

The results presented in this study provide important information regarding the diversity of bacterial species identified in fecal specimens from free-ranging long-tailed macaques (*M. fascicularis*) together with their antibiotic resistance profiles. Among the Gram-negative bacteria identified in fecal samples, *E. coli* was detected most frequently, as it was found in all (100%) of the specimens collected; this was followed by *Enterobacter* spp., which were identified in 25.71% of the specimens collected from all seven troops of macaques living in Lopburi Old Town. Fecal bacteria identified in this study exhibited high rates of resistance to common antibiotics and notably high rates of multidrug resistance; our results are similar to the previous reports of drug resistance among bacterial strains in wildlife [3,4,9,14,16,18,19,23]. The results of our study confirmed that antibiotic-resistant bacteria are prevalent in the gastrointestinal microbiota of free-ranging *M. fascicularis* in this region of Thailand.

Comparatively few fecal samples contained *Salmonella* Group B and *Citrobacter* spp.; these species were found in 1.71% and 0.57% of the specimens collected, respectively. Salmonellosis is a major foodborne illness associated with both acute and chronic diarrhea in humans as well as in domestic and wild animal hosts. Captive and free-range wildlife may serve as reservoirs for *Salmonella* spp., which can be transmitted to other wildlife, domestic animals, and humans mainly through asymptomatic carriers shedding the microbe in their feces [7]. In a previous study, *Salmonella* spp. were detected in 13.32% of fecal samples collected from wild rhesus macaques in China [6]. Similarly, *Citrobacter* spp. is a common cause of gastroenteritis in numerous mammalian hosts. *Citrobacter* spp. isolated from human and food sources have diverse properties and varying degrees of virulence and antibiotic resistance profiles. Contaminated food may be an important means for transmission of *Citrobacter* spp. to humans [28]. *Citrobacter* spp. were identified in 15% of fecal samples from wild animals; cephalosporin-resistant genes have been isolated from this species [29]. Considering the known impact of zoonotic pathogens, the presence of both *Salmonella* spp. and *Citrobacter* spp. in fecal samples of macaques living together with humans in this region represents a real and significant risk to local residents and tourists. Zoonotic infection might result from direct exposure to feces or to materials and environments contaminated with fecal materials; this may include the walls of the buildings and archaeological sites that are among the main habitats of long-tailed macaques in the Lopburi Old Town. Public policy should be designed to minimize the risk of infection by encouraging frequent hand washing, most notably after potential exposure to fecal material or to contaminated environments.

In general, *E. coli* is commonly found in the intestinal tract of mammals and birds and throughout the environment. Isolates can range in virulence from non-pathogenic to highly pathogenic. *E. coli* plays a dominant role in the evolution and transmission of antimicrobial resistance; a wide variety of resistant strains have been identified in humans, animals, food products, and the environment [3,12,20]. In this study, a high percentage of the *E. coli* isolates was resistant to common antibiotics, including amoxicillin, cephalexin, clindamycin, and erythromycin; multidrug resistance was also observed. A previous study focused on antimicrobial resistance in wildlife in England revealed that patterns of multidrug resistance were varied, although 30% of the bacterial species isolated were resistant to colistin [19]. Likewise, 85% of *Enterobacteriaceae* isolated from humans in Cameroon were resistant to amoxicillin [15] as were 94% of *E. coli* isolates in our study. However, the emergence and evolution of antimicrobial resistance among microbes in the intestinal microbiome of long-tailed macaques living in an urban ecosystem should be explored further.

Ingestion of antibiotic residues through food consumption might expose intestinal microbiota to these agents at levels exceeding the threshold concentration; these conditions would serve to favor the growth of resistant strains [11]. The nature of the intestinal microbiota is directly related to host diet and habitat [4,8]. Furthermore, anthropogenic activities have a considerable impact on the antibiotic resistance of gut bacterial communities [16,19]. Our results suggest that antimicrobial-resistant bacteria identified in feces from free-ranging might result from direct or indirect contact with anthropogenic sources such as human food, vegetables, fruit, and/or pollution in the environment from human wastes and/or with wastes contaminated with antimicrobial residues.

## Conclusion

The present study focused on an investigation of the identity and antibiotic resistance profiles of bacterial species isolated from free-ranging long-tailed macaques in Lopburi Old Town. Among our findings, 93% of *E. coli* isolates were multidrug-resistant (i.e., resistant to amoxicillin, cephalexin, clindamycin, and erythromycin) as were 83% of *Citrobacter* spp. (resistant to cephalexin, clindamycin, and erythromycin). Furthermore, 94% of *Salmonella* Group B isolates were resistant to both clindamycin and erythromycin. The present study includes basic information that may be used by clinicians for the selection of empirical therapies and likewise provides an assessment of the potential antibiotic resistance problem in Lopburi Old Town, Thailand. Strategies designed to minimize the risk of disease transmission between humans and the macaque population would benefit both human health and primate conservation efforts. Handwashing after direct or indirect contact with macaques should be actively encouraged among both visitors and local inhabitants to reduce the risk of foodborne zoonotic diseases. Enhancing public awareness of the problems associated with antimicrobial resistance may also help to limit antibiotic misuse and overuse among members of the general population and likewise may prompt efforts designed to reduce unnecessary use of antimicrobials in the livestock industry. Finally, public focus on improved methods for the treatment of wastes from both humans and the livestock industry may serve to address this problem.

## Authors’ Contributions

DB, ST, NJ, and PS designed the study and drafting the manuscript. DB and NJ performed all the experimental procedures. DB, ST, and PS conducted data analysis and interpretation. All authors read and approved the final manuscript.
